# Facing unemployment: study protocol for the implementation and evaluation of a community-based intervention for psychological well-being promotion

**DOI:** 10.1186/s12888-017-1416-x

**Published:** 2017-07-19

**Authors:** Ana Virgolino, Maria João Heitor, Joana Carreiras, Elisa Lopes, Simon Øverland, Steffen Torp, Dora Guðmundsdóttir, José Pereira Miguel, M. Fátima Reis, Osvaldo Santos

**Affiliations:** 1 Universidade de Lisboa, Faculdade de Medicina, Instituto de Medicina Preventiva e Saúde Pública, Instituto de Saúde Ambiental, Av. Professor Egas Moniz, 1649-028 Lisboa, Portugal; 2 Departamento de Psiquiatria e Saúde Mental, Hospital Beatriz Ângelo, Loures, Portugal; 3 Division of Mental and Physical Health, Norwegian Institute of Public Health, Box 4404 Nydalen, N-0403 Oslo, Norway; 4 Department of Psychosocial Science, University of Bergen, Bergen, Norway; 5 University College of Southeast Norway, PO Box 2230, N-3103 Tønsberg, Norway; 6 Centre of Public Health Sciences, University of Iceland, Reykjavik, Iceland; 7 The Directorate of Health in Iceland, Stapa vid Hringbraut, Reykjavík, Iceland

**Keywords:** Psychological well-being, Mental health promotion, Unemployment, Economic crises, Randomized field study, Study protocol

## Abstract

**Background:**

Economic crises and unemployment have profound impact on mental health and well-being. Main goal of the Healthy Employment (HE) project is to enhance intersectoral actions promoting mental health among unemployed, namely through the implementation and effectiveness-evaluation of short-term and sustainable group interventions.

**Methods:**

The project follows a RE-AIM-based (Reach, Effectiveness, Adoption, Implementation and Maintenance) framework for assessing a cognitive-behavioural and psychoeducational intervention that has been developed for promoting mental health among unemployed people. It is a short-term group intervention (five sessions, four hours each, 20 unemployed persons per group) focused on mental health literacy, interpersonal communication and of emotional regulation. Implementation of the intervention will be carried out by clinical psychologists, following a standardized procedure manual. Effectiveness will be assessed through a randomized field study with two arms (intervention and control). Participants are unemployed people (18–65 years old, both genders, having at least nine years of formal education) registered at public employment centres from different geographical regions for less than 12 months (including first-job seekers). Allocation to arms of the study will follow a random match-to-case process, considering gender, age groups and educational level. Three moments of evaluation will occur: before intervention (baseline), immediately after its ending and three months later. Main outcomes are mental health literacy, mental health related personal and perceived stigma, psychological well-being, satisfaction with life and resilience. Intention-to-treat and per-protocol analyses will be conducted. Cohen’s d coefficient and odds ratio will be used for assessing the size of the intervention effect, when significant.

**Discussion:**

Scientific and clinical knowledge will be applied to promote/protect psychological well-being of unemployed people. While the first phases of the project are funded by the European Economic Area Grants, long-term assessments of the intervention require a larger timeframe. Further funding and institutional support will be sought for this purpose. Already established intersectoral collaborations are key-assets to reach long-term sustainability of this project.

**Trial registration:**

The study was registered with the Australian New Zealand Clinical Trials Registry; Prospectively registered number: ACTRN12616001432404; date of registration: 13 October 2016.

## Background

The recent European financial crisis has affected large proportions of the population in many countries, most particularly in Greece, Ireland and Portugal [1–3]. In Portugal, due to the assistance received from the European Financial Stabilization Mechanism, several harsh austerity measures were adopted, leading to budget cuts across different areas including the health sector [2]. This led to a reduced health system capacity, jeopardizing the implementation of health promoting actions and prevention programs [1, 4].

Economic crises present numerous potential challenges to mental health, such as reduced access to health care services [1, 5, 6] and deterioration of individuals’ psychological well-being as a result of job loss. Several studies have shown that in a context of recession or adverse economic climate, those affected by job loss have an increased risk of suffering from psychological disorders, including depression, anxiety, alcohol and drug abuse [5, 7, 8]. In Portugal, the unemployment rates have increased over the last 10 years. In late 2014, when the funding protocol for this project was first submitted (European Economic Area [EEA] Grants), the unemployment rate in Portugal was 13.5%. In February 2016, it was slightly lower (12.3%), but still higher than the European Community average (10.3%) [9]. Santana et al. [10] reported that the decreasing tendency of suicide rates observed between 1989 and 1993 and 1999–2003 (−5.4%) was inverted in 2008–2012 (with an increase of 22.6%).

Extensive work has been done to identify the complex links between economic downturns and suicide [11–14] or mood disorders [5, 8, 15]. For example, higher levels of rurality and material deprivation have been identified as main determinants of suicide in Portugal, in the context of the recent financial crisis [10]. Anyway, results remain conflicting. For example, it has been argued that difficult economic contexts do not necessarily imply increased adverse health (mortality rates can even fall), namely because economy slowdowns may favour healthier behaviours (e.g., as increased sleep time and physical activity, reduction of unhealthy foods and/or alcohol intake and less risky driving) [16]. Also, the relationship between unemployment and suicide is possibly moderated by available welfare-system support and expectancies of regaining meaningful employment, which are conditions that vary over time and between countries [17–19].

Professionally active-aged individuals may change between a variety of dynamic employment-related forms, ranging from unemployment or underemployment to employment or even overemployment [15, 20]. Deleterious effects of unemployment extend beyond the individual, affecting also relational, family and professional realms [21–23]. Under this last aspect, when attempting to make the transition back to paid employment, individuals can lack required or up-to-date skills, which can lead to precarious employment and increase risk of returning to unemployment [5, 7, 24–26].

The negative health effects of unemployment or underemployment can be attenuated or prevented through the implementation of wide restructuring health promotion actions [1, 27]. Psychological well-being depends upon a complex and interrelated set of individual, socioeconomic and environmental factors [28, 29]. Efficacious mental health protection and promotion actions need to involve several community domains and societal sectors (both public and private) such as primary health care services, social welfare support and promotion of active labour market programs [30, 31].

Several programs focused primarily on building capacity, resilience and enhancement of job search-related skills have been developed in different countries. Such intervention programs have revealed short and long-term positive effects in protecting psychological well-being among those unemployed [24, 32–36].

In Portugal, the weight of mental health related problems within the global burden of diseases, as well as their direct and indirect costs, are only marginally recognized [37, 38]. As a consequence, there is a clear deficit of evidence-based large-scale promotion mental health programs [39]. Indeed, in contrast to what has happened in other countries that implemented evidence-based programs to face unemployment rate increase [5, 31], only incipient and rather delayed responses have been developed to protect psychological well-being of unemployed individuals [3].

**Fig. 1:**
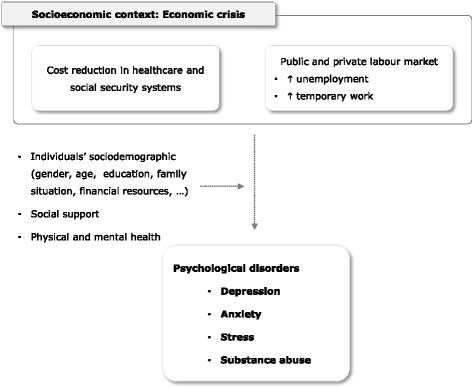
General conceptual framework of the HE project

For these reasons, this community intervention project, the Healthy Employment (HE), has been developed to contribute to the protection and promotion of psychological well-being for the unemployed. The project includes policy, organizational and individual capacity-building opportunities for both the health sector and adjacent non-health sectors, by facilitating mental health impairment early-detection and intervention in individuals with signs of psychological suffering [40, 41] (Fig. 1).

Main products within the HE project context are the implementation and evaluation of a Portuguese-specific community intervention for psychological well-being promotion among unemployed individuals, promoting mental health and preventing negative health effects of economic and social adverse contexts. In order to be feasible and sustainable at long-term, the intervention was defined as low-cost and standardized. Also, the project endorses a systemic approach by enhancing socio-cognitive and mental healthcare referral skills among professionals in public employment centres.

### Aims and objectives

The main goal of this study is to implement a community-based intervention for psychological well-being protection and promotion and to assess its short-term effectiveness. Specific objectives are:

(i) to implement a standardized intervention for psychological well-being promotion among Portuguese facing unemployment in two different community settings (rural versus urban settings)
(ii) to assess the effectiveness of this mental health promotion intervention in terms of psychological well-being and resilience in job-searching.

## Methods/design

### Intervention description

Previous good practices (from different countries) [34, 42] and the opinion of a panel of Portuguese experts was used for the elaboration of a Portuguese cultural-specific adapted model of intervention aiming at the psychological well-being protection and promotion. More specifically, the intervention focuses on the promotion of unemployed individuals’ social, emotional and interpersonal skills. It also targets the strengthening of resilience and of the ability to overcome the adversity of unemployment, by maximizing the personal use of owns’ resources for employability enhancement and future success in re-employment. It is a short-term group intervention (around 20 h, delivered in a two- to three-weeks period) and follows a cognitive-behavioural and psychoeducational paradigm (focusing on mental health literacy). Strategic skills to be promoted are: reduction of mental health related stigma, recognition of psychological suffering (depression and anxiety), coping with depression and anxiety, effective communication and emotional regulation. Regarding the format and structure of the intervention, a maximum of five sessions, three hours each (maximum: 20 persons per group) will be followed.

The intervention was standardized (procedure manual with training and implementation material for facilitators), covering the following modules: Mental health literacy (about depression, anxiety, burnout, emotions), work-life balance, mental health related stigma (personal and perceived stigma), assertiveness training and definition of individual plans of action (defining short, medium and long term goals). Procedures of the intervention will include: roleplays, group discussion of vignettes, emotional and cognitive self-awareness, individual tasks based on cognitive-behavioural intervention (e.g., ABC task for assessing emotions, behaviours and cognitions; self-assessment assertive behaviour grill, for assessing contexts of assertiveness-related difficulties, etc.).

### Study design

The assessment of the intervention will be done according to the following RE-AIM (Reach, Effectiveness, Adoption, Implementation and Maintenance) framework [43] dimensions: reach, effectiveness, implementation and maintenance of qualities/properties.

The project’s reach assessment will be evaluated by measuring the proportion of invited users from two public employment centres that accept to participate in the intervention.

**Fig. 2:**
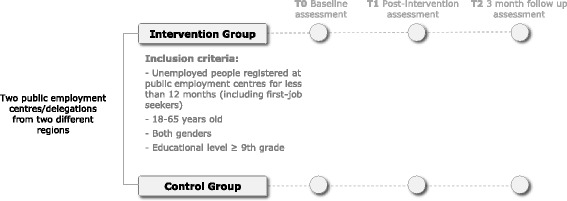
Community-based randomized field: study design

Effectiveness will be assessed through a randomized experimental field study with two arms (intervention group and control group) (Fig. 2). The intervention group will participate in the short-term cognitive-behavioural and psychoeducational sessions; the control group will receive the care-as-usual from public employment centres.

The implementation assessment will be done by evaluating the extent to which the syllabus and training intervention is adequate and is accomplished across the intervention (fidelity assessment). A specific fidelity assessment form will be filled in by trainers and co-trainers (based on already existent toolkit forms) [44]. Also, trainees’ satisfaction with practical exercises will be assessed.

Finally, managers/supervisors’ willingness to replicate the intervention in other units of public employment centres will be assessed as a measure of the extent to which the intervention will be sustained over time (maintenance assessment).

### Sampling aspects

The target population of the HE intervention are unemployed people registered at public employment centres for less than 12 months (including first-job seekers). Random recruitment of participants (for both arms of the study) occurs in two employment centres (from different geographical regions), with match-to-case allocation in arms according to gender, age groups (18–24, 25–44, and 45–65 years old) and educational level (9 to 12, and more than 12 years of completed school – higher education). The full list of the two public employment centres users (with inclusion criteria) will be stratified by gender, age groups and educational level. In each combined stratum, random allocation will be done to each participant who accepts to participate.

Inclusion criteria: unemployed users from public employment centres, 18 to 65 years old, both genders, having at least nine years of formal/public education and being registered in the selected public employment centres at the moment of recruitment.

Exclusion criteria: public employment centres’ unemployed users already attending, at the moment of the recruitment, other public employment centre’s courses, or users with a diagnosed mental or physical severe or incapacitating disease or disability.

Two public employment centres/delegations from two Portuguese regions will be selected (intentional sampling) and invited to participate in the project. The involved public employment centres/delegations differ in terms of size (big dimension vs. small dimension) and type of region (rural population vs. urban population). In each centre/delegation, participants are randomly selected from the full database of users. Allocation to arms is done also in a random way, though following gender, age and educational level strata inter-arms matching.

In each of these settings, the initial (at baseline) users sample size will be of 50 participants with 50 other participants as controls. Previewing a dropout rate of 30% till the end of the project, we estimate to have a final users’ sample size of 35 participants in each of the arms, in each employment centre (70 participants per arm, in total). With this sample size, we expect to have 80% power to detect an effect size of 0,48 in mean scores of mental health literacy, psychological well-being, satisfaction with life and resilience [45].

### Variables in study

Data collection for the controlled community sample will be done for both arms of the trial on three different moments: before the intervention (at baseline), immediately after its ending and three months later.

The indicators for the intervention effectiveness will be: mental health literacy, mental health related personal and perceived stigma, psychological well-being (depression and anxiety), satisfaction with life and resilience. Secondary outcomes include opinions and satisfaction with intervention by intervened unemployed, rate of participation and completion of the intervention. The socio-demographic variables that will be used to characterize the sample and for adjusting outcomes assessment include: age, gender, educational level, previous professional activities, having or not dependent relatives (children, dependent elderly or other relatives to care) and household overall financial comfort.

All questionnaires measuring/assessing constructs (i.e., latent variables) were selected on basis of their psychometric qualities (e.g., internal and temporal reliability, validity) when applied to adult Portuguese general populations. Table 1 provides an overview of the validated instruments that will be included in the effectiveness assessment. Taking into consideration the time usually reported for filling in each of the selected instruments, it is estimated that the total time for filling the complete battery of questionnaires is around 20 min. We will run a pilot test with at least 10 unemployed persons to assess the burden of the questionnaires.

**Table 1:** Instruments used in the effectiveness assessment

Assessed constructs	Questionnaire / measure scale	Description
Mental health literacy	Modified/shortened versions of the following instruments:	The DSS is designed to measure stigma associated with depression. It has two subscales which measure two different types of stigma: personal and perceived. Responses to each item are measured on a five-point scale (ranging from zero ‘strongly disagree’ to four ‘strongly agree’). Higher scores indicate higher levels of depression stigma.
	• Depression Stigma Scale (DSS)	
	• Depression Literacy Questionnaire (D-Lit)	
		The D-Lit assesses mental health literacy specific to depression. The questionnaire consists of 22 items which are true or false. Respondents can answer each item with one of three options – true, false or don’t know. Each correct response receives one point. Higher scores indicate higher mental health literacy of depression.
	• Anxiety Literacy Questionnaire (A-Lit) [46]	
		The A-Lit assesses mental health literacy specific to anxiety. The questionnaire consists of 22 items which are true or false. Respondents can answer each item with one of three options – true, false or don’t know. Higher scores indicate higher mental health literacy of anxiety.
		For each of these scales, we will use the items that will be most adequate to assess literacy gains, according to the contents that will be worked out during the intervention.
Psychological well-being (depression and anxiety)	General Health Questionnaire (GHQ) [47, 48]	The General Health Questionnaire (GHQ) is a screening scale for identifying minor psychiatric disorders in the general population and within community or non-psychiatric clinical settings. GHQ assesses the respondent’s current state and asks if that differs from his or her usual state.
	Brief Symptom Inventory (BSI) [49]	The Brief Symptom Inventory is a 53-item self-report inventory in which participants rate the extent to which they have been bothered (0 = “not at all” to 4 = “extremely”) in the past week by various symptoms. The BSI has nine subscales designed to assess individual symptom groups: somatization, obsessive-compulsive, interpersonal sensitivity, depression, anxiety, hostility, phobic anxiety, paranoid ideation, and psychoticism. The BSI also includes three scales that capture global psychological distress.
Satisfaction with life	Satisfaction with Life Scale (SWLS) [50]	The SWLS is a short 5-item instrument designed to measure global cognitive judgments of satisfaction with one’s life.
Resilience	Connor-Davidson Resilience Scale (CD-RISC) [51]	The CD-RISC is a 25 item scale created to address aspects of resilience and for use in clinical practice.

### Data analysis

Statistical analysis will be carried out using IBM/SPSS 21.0 (or a more updated version, at the moment of the analysis) and R packages. For all tests, the level of statistical significance will be set as α = .05.

For the effectiveness assessment, socio-demographic and outcome variables will be described for each moment of observation. Two strategies of data analysis will be done: (1) intention-to-treat (ITT), as a primary analysis, and; (2) per-protocol, as a supportive analysis. The ITT analysis (treatment as assigned approach), will consider all participants randomly allocated to each arm of the study at baseline with a last observation carried forward imputation for those who did not undergo the final evaluation. The per-protocol analysis (treatment as received approach), will include only the subset of the ITT sample who will completed the evaluations without any major protocol violations. When describing differences between intervention and control group, results from both types of analysis will be reported; for effect size assessment, only per-protocol approach results will be reported, in order to have an estimation of the impact of the treatment as received.

Univariate description of variables will be summarized by central tendency measures (means and medians, according to the normality of their distributions) and respective dispersion measures (standard deviation and range). Normality of data will be assessed through the analysis of kurtosis and skewness.

Association between nominal variables will be assessed through chi-square test (with Yates adjustment, when necessary). Continued variables will be compared (between arms, at the same momentum) by independent samples t-student test or by its non-parametric equivalent (Mann-Whitney test), when adequate.

Effect of the intervention will be studied by linear or logistic regression analysis (according to the nature of the dependent variable), having as dependent variables test-retest variations and adjusting for conceptually (and statistically) relevant variables. Model assumptions will be tested by analysis of residuals and influence diagnostics through Cook’s distance. Cohen’s d coefficient and odds ratio will be used for assessing the size of the intervention effect, when significant.

### Ethics

This intervention protocol follows the code of ethics of the Declaration of Helsinki [52]. All participants will be asked to sign an informed consent, with detailed information concerning the goals and procedures of the project, as well as with regard to their full right to refuse or quit their participation at any time. The written informed consent will be signed before the first intervention session.

If the intervention proves to be effective, participants enrolled in the control arm will be invited to benefit from it as well. This research protocol was approved by the Portuguese Protection Data Authority (CNPD). Ethics approval was obtained from the Ethical Committee of Academic Centre of Medicine of Lisbon (University of Lisbon).

## Discussion

In a context of adverse economic climate, vulnerable groups experiencing unemployment have an increased risk of suffering from mood disorders and psychological distress [5, 53, 54]. This paper describes a community-based randomized field study protocol for assessing the effectiveness of a psychological well-being protection and promotion intervention, aiming to build capacity and reduce inequalities within the context of unemployment. This intervention is foreseen as strategic due to its applicability to different community settings and targeted populations. Moreover, the intervention has also a component of socio-cognitive skills development and training for an adequate recognition of psychological suffering and referral to primary healthcare and mental health services.

A main challenge of the field experiment concerns some fundamental characteristics of the intervention, namely in terms of format and duration. The intervention is currently planned to follow a short-term group format, maximizing its potential to be adopted, replicated and sustained in different settings (in both public and private sectors). Another difficulty relates to the fact that the population that may benefit from the intervention is highly heterogeneous, namely regarding financial, sociocultural backgrounds and level of psychological suffering. Therefore, the added-value of the intervention for participants will vary substantially and the overall trial results will need to be stratified by individuals’ characteristics.

Considering the timeframe of the EEA Grants 2009–2014 that financially supported this project (see acknowledgements/funding section), the final evaluation of the intervention will be done three months after its ending. This is a limitation of this study protocol because three months might be a narrow time frame for detecting mental health promotion effects and, on the other hand, possible intervention-related changes can be temporary and not sustainable. Nevertheless, if the intervention shows positive results at the last observation moment, the project team will apply for new funding opportunities to follow the recruited cohort (within this trial) for a longer period.

The external validity of the study will also require replication with larger numbers of participants. The expected results of the study, together with the fact that the involved partners include multilevel and intersectoral stakeholders, in the areas of unemployment, temporary employment, social security, local authorities, health services and entrepreneurial activities, will be instrumental to inform decision makers.

This project is embedded in the strengthening of the bilateral relations between Portugal and EEA Grants’ Donor States (Norway, Iceland and Lichtenstein), namely by sharing scientific knowledge about mental health promotion of vulnerable groups such as those unemployed. The tangible joint results that we hope to reach through this opportunity of cooperation will reinforce the political, professional and human ties between these countries.

## Abbreviations

A-Lit: Anxiety Literacy Questionnaire
BSI: Brief Symptom Inventory
CD-RISC: Connor-Davidson Resilience Scale
CNPD: Portuguese Protection Data Authority
D-Lit: Depression Literacy Questionnaire
DSS: Depression Stigma Scale
EEA Grants: European Economic Area Grants
GHQ: General Health Questionnaire
HE: Healthy Employment
ITT: Intention-to-treat
RE-AIM: Reach, Effectiveness, Adoption, Implementation and Maintenance
SWLS: The Satisfaction with Life Scale
